# Fretting and Corrosion in Modular Shoulder Arthroplasty: A Retrieval Analysis

**DOI:** 10.1155/2016/1695906

**Published:** 2016-06-28

**Authors:** Johannes A. Eckert, Ulrike Mueller, Sebastian Jaeger, Benjamin Panzram, J. Philippe Kretzer

**Affiliations:** Laboratory of Biomechanics and Implant Research, Clinic for Orthopedics and Trauma Surgery, Heidelberg University Hospital, Schlierbacher Landstrasse 200a, 69118 Heidelberg, Germany

## Abstract

Tribocorrosion in taper junctions of retrieved anatomic shoulder arthroplasty implants was evaluated. A comparison of the tribocorrosion between cobalt-chromium and titanium alloy stems was conducted and the observations were correlated with the individual's clinical data. Adverse effects caused by metal debris and subsequent elevated serum metal ion levels are frequently reported in total hip arthroplasty. In total shoulder arthroplasty, to date only a small number of retrieval analyses are available and even fewer address the issue of tribocorrosion at the taper junctions. A total of 36 retrieved hemiarthroplasties and total shoulder arthroplasties were assessed using the modified Goldberg score. The prevalence of fretting and corrosion was confirmed in this cohort. Titanium stems seem to be more susceptible to damage caused by tribocorrosion than cobalt-chromium stems. Furthermore, stemless designs offered less tribocorrosion at the taper junction than stemmed designs. A weak correlation between time to revision and increased levels of tribocorrosion was seen. Whether or not tribocorrosion can lead to adverse clinical reactions and causes failure of shoulder arthroplasties remains to be examined.

## 1. Introduction

Shoulder arthroplasties for primary osteoarthritis of the shoulder are used in steadily increasing numbers [1, 2] with good results [3, 4]. Historically, monoblock designs have been used. As interindividual anatomy of the glenohumeral joint varies immensely, modular designs have been established. Modular implant designs are well known in the hip, where they allow for an optimal restoration of biomechanics. In hip arthroplasty damage at the modular taper connection has been described as a cause for postoperative complications like the so-called trunnionosis [5, 6]. This complication is caused by corrosion and a release of metal debris. Consequently, tribocorrosion can lead to local and, in extreme cases, systemic reactions [7]. Although the effect of head-neck taper junction is generally considered to be benign, some authors describe the percentage of complications in hip replacements caused by corrosion to be as high as 20%; for certain designs some studies describe up to 30% revision rate [8]. In hip arthroplasty corroded tapers often present surface irregularity like fretting scars, worn areas, pits, and etch marks [9, 10]. In this regard several different factors are associated with tribocorrosion, including material combination, head size, offset, implantation time, and flexural rigidity [7]. Whereas multiple retrieval studies regarding hip implants are available, only a small number of retrieval studies for modular shoulder arthroplasty exist [11, 12].

In shoulder arthroplasty common stem materials are cobalt-chromium alloys (CoCr) and titanium alloys (Ti). Furthermore, different shoulder arthroplasties exist with regard to stem design. Regular long stems utilize diaphyseal fixation, whereas “stemless” designs with corolla or cage screw are anchored in metaphyseal manner. There are also different materials (like Ti, CoCr, or ceramics) for the heads available.

The purpose of this study was to assess and analyze tribocorrosion of modular taper junctions of the retrieved shoulder arthroplasty implants and describe them with regard to severity, extent, and frequency. Tribocorrosion was compared in mixed metal (head: CoCr, stem: Ti) and the same metal (head and stem: CoCr) implants as well as in stemmed and stemless fixation. It was hypothesized that there is a higher incidence of corrosion in mixed metal implants whereas no difference regarding stem fixation was expected. Furthermore, it was planned to correlate the findings with clinical data and to assess whether increased tribocorrosion causes earlier failure of anatomic shoulder arthroplasty.

## 2. Materials and Methods

### 2.1. Epidemiology

**Figure 1:**
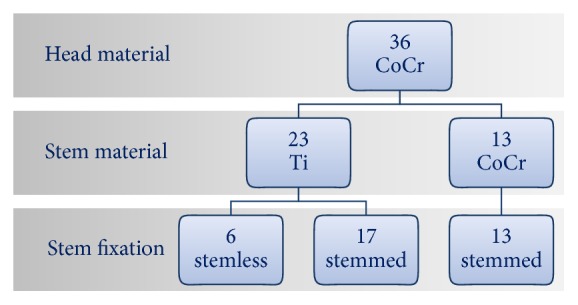
Distribution of material and fixation among the retrieved components.

**Figure 2:**
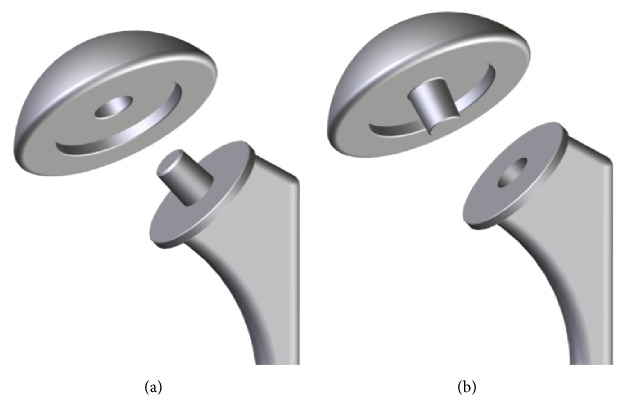
Analyzed types of shoulder implants: two different stems types were analyzed: retrieved stems had either a male (a) or a female (b) taper.

A total of 38 consecutively retrieved anatomic implants were available for analysis. All explants were revised at the Clinic for Orthopedics and Trauma Surgery of the Heidelberg University Hospital. Two of the retrieved implants had a Ti head and a ceramic head, respectively, which were excluded. Out of the 36 retrieved implants, 30 had a stem fixation, whereas 6 had a stemless fixation. In all cases, CoCr heads were used. Twenty-three of the analyzed implants (64%) had a Ti stemmed or stemless fixation, and 13 implants (36%) featured a CoCr stemmed or stemless fixation (Figure 1). All implants were used in anatomical total shoulder arthroplasty (TSA; *n* = 7) or hemiarthroplasty (HA; *n* = 29). The mean time to revision was 3.7 ± 4.1 years (0.03–13.5 years), 10 patients were male, and 27 patients were female. Manufacturers included Tornier (*n* = 14), Zimmer (*n* = 7), Arthrex (*n* = 7), Depuy (*n* = 3), Biomet (*n* = 2), Exactech (*n* = 1), Plus Orthopedics (*n* = 1), and Synthes (*n* = 1). In 4 cases, the stem had a female taper, whereas, in all the other cases, the stem had a male taper (Figure 2). Among the four female tapers, three were made of CoCr and one of Ti. All the stemless implants had a male taper on the humeral component. Patient demographics are given in Table 1. The reasons for revision and distribution are given in Table 2. Inclusion criteria were as follows: explantation of the entire humeral component and availability of all clinical data (dates of primary surgery/revision surgery, age, body weight, body mass index (BMI), and indication for revision).

### 2.2. Qualitative Damage Assessment

Tribocorrosion was graded on a scale from 1 to 4 depending on the extent and the magnitude of the damage as described by Goldberg et al. [9] and modified by Cusick and colleagues [11] (Table 3). This classification is the most commonly used damage scoring system to identify tribocorrosion on retrieved implants. The taper interfaces were macroscopically evaluated by two independent observers (JAE, UM). Any damage caused during implantation and explantation, respectively, was excluded from the assessment. Both male and female tapers were observed; hence, for each implant, a total of 2 scores were obtained: one for the stemmed/stemless fixation and one for the head. No postoperative cleaning procedure was performed on the components in order to avoid removal of corrosion products. As superficial corrosion products might cover fretting marks, a real distinction between fretting and corrosion is difficult to be achieved macroscopically. Therefore, the term “tribocorrosion” was chosen.

### 2.3. Statistics

The interrater reliability between both observers was evaluated using kappa statistics and the score of the primary observer (JAE) was used for statistical analyses. Furthermore, the intrarater reliability was calculated based on 13 samples for one observer (JAE). Descriptive statistics were calculated for all measurements.

A Shapiro-Wilk test revealed a nonnormal population for the study cohort (*p* = 0.05); hence the nonparametric Mann-Whitney* U* test was conducted to test for statistical significance. To analyze correlations between tribocorrosion and clinical data, the Spearman rank correlation coefficient was used. A value of *p* < 0.05 was considered statistically significant. All statistical analyses were performed using SPSS software (version 23.0; SPSS Inc., Chicago, IL).

## 3. Results

### 3.1. Reliability

Cohen's kappa statistic revealed agreement between both observers in any case (*p* < 0.001). Substantial strength of agreement was found for the stem (*κ* = 0.682), whereas the agreement for the head (*κ* = 0.553) was moderate.

The intrarater reliability tested with Cohen's kappa showed a substantial agreement for the stems and the heads, respectively (*κ* > 0.750, *p* < 0.001; JAE).

### 3.2. Assessment of Tribocorrosion

**Figure 3:**
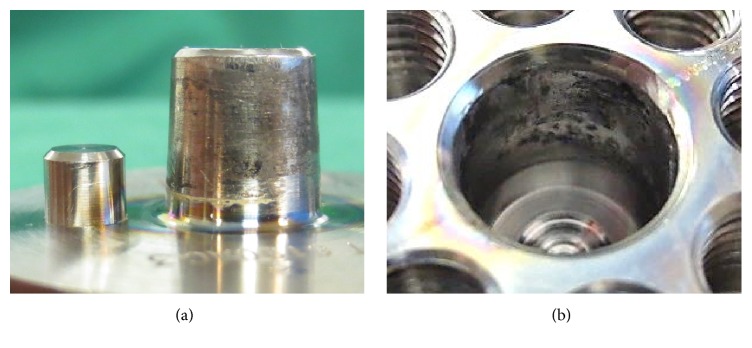
Severe tribocorrosion on male (a) and female (b) taper of a retrieved prosthesis. The time to revision was 9.2 years.

**Figure 4:**
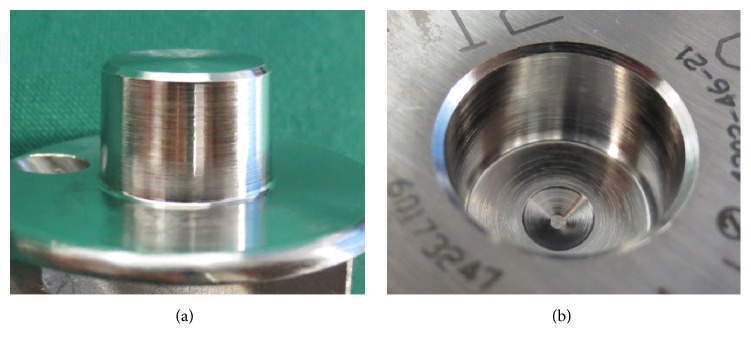
Minimal tribocorrosion on male (a) and female (b) taper of a retrieved prosthesis. This prosthesis was implanted for 8.8 years.

Tribocorrosion (score ≥ 2) was present on 27 of the 36 heads (75%) and 29 of the 36 stems (81%). Seven of the 36 implants (19%) showed at least moderate tribocorrosion (score ≥ 3 for both tapers), three of which (8%) showed severe tribocorrosion (score = 4 for both tapers). One of the severely affected cases is shown in Figure 3 and one of the minimally affected implants is seen in Figure 4.

**Figure 5:**
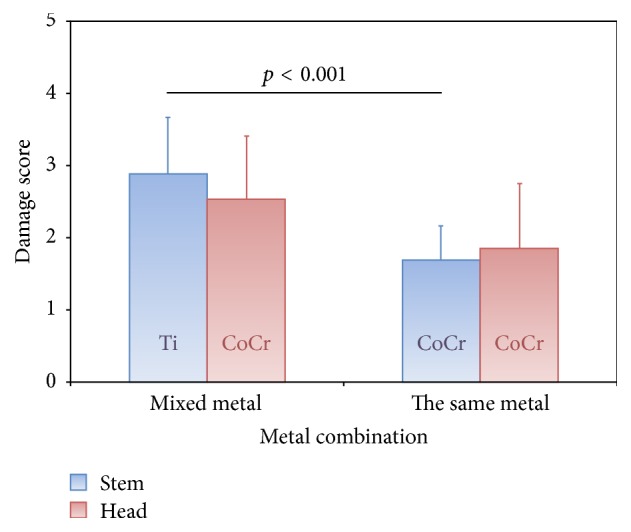
Comparison of the damage scores for the head and stem taper depending on stem material. Heads are all made of CoCr.

Significantly greater tribocorrosion (*p* < 0.001) was seen in mixed metal combinations where Ti stems were used (2.88 ± 0.78) compared to the same metals using CoCr stems (1.69 ± 0.48). For the CoCr heads there was a tendency of increased tribocorrosion when combined with Ti stems (2.53 ± 0.87) compared to the combination with CoCr stems (1.85 ± 0.90), although this difference was not statistically significant (*p* = 0.072; Figure 5).

**Figure 6:**
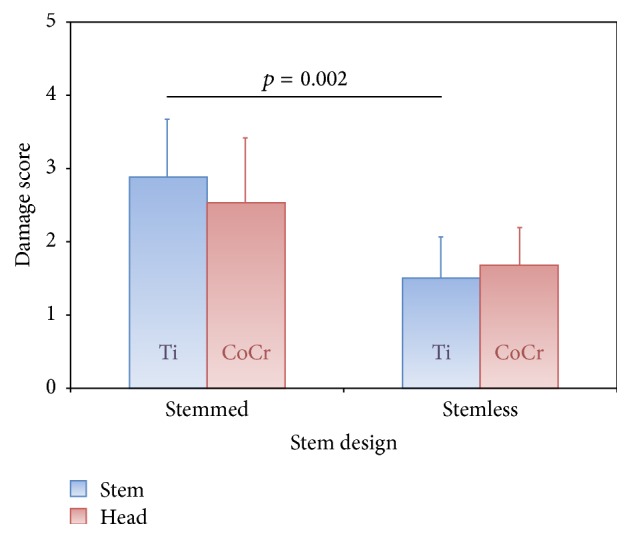
Comparison of the damage scores for the head and stem taper depending on stem design. Only titanium stems are included.

Of the 36 retrieved implants, 30 (83%) had a stemmed and 6 (17%) a stemless fixation. All stemless implants were made of Ti alloy. In designs with a stem, the stem material was Ti in 17 (57%) cases and CoCr in 13 (43%) cases. To compare the effect of the stem fixation, only Ti tapers were included (Figure 1). The stemmed designs showed significantly higher tribocorrosion (*p* = 0.002) for the stem tapers (Figure 6). Time to revision of stemless (2.3 ± 1.4 years) and stemmed (3.4 ± 3.6 years) designs was comparable. For the head tapers, stemless designs showed a tendency for less tribocorrosion (*p* = 0.052), although the differences were not statistically significant.

### 3.3. Correlations with Clinical Data

Correlations between clinical data and the observed damage scores were evaluated (Table 4). Increased tribocorrosion was seen in retrieved implants with a longer period* in situ*; however, the correlation was weak (*R* = 0.460, *p* = 0.005). Apart from that, no correlation with clinical data was found.

## 4. Discussion

In this retrieval study, tribocorrosion was seen in the majority of the retrieved implants. However, only a small subset of 16% showed moderate to severe corrosion for both tapers; most prostheses featured only mild tribocorrosion. Generally, there was a higher incidence of tribocorrosion in mixed metal implants as hypothesized although it was only significantly higher for the stem components. Interestingly, retrieved implants with Ti stems showed greater corrosion than the CoCr stems. Furthermore, stemmed Ti implants exhibited increased corrosion scores than stemless Ti implants. Therefore, the hypothesis regarding the fixation has to be rejected.

Only two other retrieval studies on tribocorrosion in shoulder arthroplasty have been published [11, 12] and only one of those examined anatomic implants. Teeter and colleagues [12] also reported tribocorrosion in their cohort, albeit at a much lower level (38% of the stems and 32% of the heads, compared to 81% and 75%, resp., as found in this study). They found tribocorrosion to be only prevalent in stemmed designs, whereas no tribocorrosion was seen in the stemless implants. In the current study a similar tendency was observed; however, some tribocorrosion was also found in the stemless implants. The effect of material combinations was not compared in the study by Teeter et al. [12]. The differences regarding the severity of corrosion might be explained by variations in the retrieval cohorts.

With the establishment of modular junctions in endoprostheses in the 1980s, numerous studies were published analyzing the risk of corrosion for modular hip prostheses. As early as 1991, Mathiesen and colleagues described corrosion in a cohort of retrieved hip implants, with the junctions between the modular components being regarded as the source for corrosion processes [13]. Corrosion processes of various kinds are seen in 10 to 40% of retrieved implants [9, 14–17]; some authors reported the rate of corrosion to be as high as 84% in their retrieval cohort [18] (nearly matching the 81% found in this study). Most authors described a risk of metal ion and particle release associated with tribocorrosion at taper junctions and, thus, a higher risk for third-body wear, particle-induced osteolysis, and aseptic loosening.

Possible reasons for the vulnerability of the taper junctions in modular prostheses have been described. For example micromotions between two components may lead to fretting and corrosion [19, 20], augmented by disruption of the passive surface oxide layer [21].

The mixed metal combination (Ti-CoCr) may exhibit higher tribocorrosion than the same metal combination because of the potential for additional galvanic corrosion. Galvanic corrosion may occur intergranularly or if metals of different electrochemical potential are combined [14, 22]. Comparable observations have been reported for mixed metal hip implants [23, 24]. Corrosion was observed in mixed metal couples (Ti-CoCr) but also in the same metal combinations (CoCr-CoCr and Ti-Ti). However, corrosion has been frequently described to be higher in mixed metal hip implants [9, 14, 16, 17].

However, in this study mixed metal implants were only available for the Ti stems in combination with CoCr heads. Thus, these findings should not be generalized for any kind of material or combination.

Stemmed implants might show higher torque levels at the modular interface due to their diaphyseal anchorage than the stemless implants which are anchored in metaphyseal manner. This might offer an explanation for the described findings of lower tribocorrosion levels for stemless implants. Also, stemless implants should only be used in patients with good bone stock in order to secure a suitable fixation in the bone. Hence, patient specific factors (like patient activity and muscle strength among others) might generally have an influence on tribocorrosion.

In the present cohort, implants with a longer time to revision exhibited slightly higher tribocorrosion levels than implants with a shorter time. This correlation, however, was only weak. Whether or not the described findings correlate with serum ion levels or clinical findings is impossible to assess in the retrospective setting of a retrieval analysis. For this purpose, additional prospective long-term studies need to be conducted. In hip prostheses, the effects of increased cobalt, chromium, and titanium levels on pseudotumor formation as well as other adverse clinical findings and subsequent revisions have been described multiple times [25–31]. In total knee arthroplasty, elevated serum metal ion levels have been described in experimental settings [32] as well as clinically [33]; however, it remains debatable whether they have any clinical implications [34]. In shoulder arthroplasty, to our knowledge no study has shown elevated metal ions or pseudotumor formation thus far. It remains unclear whether or not the tribocorrosion in shoulder arthroplasty is of similar clinical importance as it is in hip arthroplasty.

The relevance of retrieval studies has increased as they allow an assessment of the interaction between implant and patient anatomy. Furthermore,* in vitro* testing often cannot fully predict the* in vivo* behavior of implants [35, 36]. Due to regulations and quality control, retrieval management is getting more important and has become mandatory in Germany [37].

This study has some limitations: As it is a retrieval study, the design is retrospective, and as mentioned before tribocorrosion cannot be correlated to acute clinical findings such as blood results. This would be necessary to highlight adverse clinical reactions but can only be achieved in a prospective setting. The terms fretting and corrosion, while clearly defined, are used in different ways in retrieval analysis. While some authors distinguish between fretting and corrosion, we found it hard to classify the differences in this study cohort. Also, whether it actually makes a difference if an implant shows fretting or corrosion or even if corrosion might be the result of fretting is yet to be examined. Thus, the term tribocorrosion was used throughout this publication.

The subgroup of stemless implants was small (Figure 1). As such, further research is necessary to support the observation of less tribocorrosion in stemless implants. Furthermore a comparison between male and female tapers has not been performed due to the limited number of female tapers. Also, prostheses from eight different companies were included in this study. Thus, the high number of different designs and the partially low numbers for the respective prostheses make it impossible to assess the effect of design factors on tribocorrosion.

The retrieved implants were implanted for a number of reasons in a diverse study cohort that features patient ages between 45 and 86 years. Furthermore, they were revised for different reasons and do not necessarily represent a cross section of the normal shoulder arthroplasty population.

## 5. Conclusion

Tribocorrosion takes place in modular junctions of anatomic shoulder arthroplasties. In our cohort, titanium stems showed significantly more tribocorrosion than cobalt-chromium stems. Also, stemmed designs showed increased tribocorrosion than their stemless counterparts, even though stemless designs represent only a small proportion of the study population. High corrosion scores at the stem correlated with high corrosion at the head tapers. Further studies will be needed to assess clinical implications for trunnion wear in shoulder arthroplasty.

## Figures and Tables

**Table 1:** Patient demographics of the 36 retrievals.

Parameter	Value
Number of patients	36
Age, in years	68 ± 11 (45–86)
Sex	
Female	26 (72%)
Male	10 (28%)
Time to revision, in years	3.7 ± 3.9 (0.03–13.5)
Side	
Left	13 (36%)
Right	23 (64%)
BMI, in kg/m^2^	28.7 ± 5.8 (18.4–43.6)

**Table 2:** Reasons for revision and distribution.

Reasons for revision	Total	TSA	HA
Infection	9 (25%)	1 (14%)	8 (28%)
Instability	15 (42%)	2 (29%)	13 (45%)
Aseptic loosening	5 (14%)	2 (29%)	3 (10%)
Progression of osteoarthritis	5 (14%)	1 (14%)	4 (14%)
Periprosthetic fracture	2 (6%)	1 (14%)	1 (3%)

**Table 3:** The modified Goldberg score [9] according to Cusick et al. [11].

Damage	Score	Criteria
Minimal	1	Fretting on <10% of the surface and no corrosion damage
Mild	2	Fretting on >10% of the surface and/or corrosion attack confined to one or small areas
Moderate	3	Fretting on >30% of the surface and/or aggressive local corrosion attack with corrosion debris
Severe	4	Damage over the majority (>50%) of the surface with severe corrosion attack and abundant corrosion debris

**Table 4:** Spearman's correlation coefficients for the damage scores depending on clinical data (*n* = 36).

	Stem taper		Head taper	
	*R*	*p*	*R*	*p*
Time to revision	0.165	0.335	0.460	0.005
BMI	0.038	0.827	0.154	0.371
Age	0.089	0.606	−0.175	0.309
